# F104. A STANDARDISED EMPIRICAL INVESTIGATION OF THE CLINICAL PROFILE OF PSYCHOSIS FOLLOWING TRAUMATIC BRAIN INJURY (PFTBI)

**DOI:** 10.1093/schbul/sby017.635

**Published:** 2018-04-01

**Authors:** Rachel Batty, Neil Thomas, Andrew Francis, Malcolm Hopwood, Jennie Ponsford, Susan Rossell

**Affiliations:** 1 Swinburne University of Technology; 2 RMIT University; 3 University of Melbourne; 4 Monash-Epworth Rehabilitation Research Centre

## Abstract

**Background:**

Persons who experience symptoms of psychosis following a brain injury live with a complex dual diagnosis that is often accompanied by substantial distress and disability. However, a comprehensive examination of the clinical presentation of PFTBI using standardised clinical measures has not been reported in the literature. This information is vital for accurate diagnosis and effective treatment.

**Methods:**

Patients with PFTBI (n =10) and schizophrenia (n =98) participated in a comprehensive clinical assessment that included the Structured Clinical Interview for DSM-IV-TR Axis I Disorders (SCID-I/P), the Positive and Negative Syndrome Scale (PANSS), the Scale for the Assessment of Positive Symptoms (SAPS), the Scale for the Assessment of Negative Symptoms (SANS), and the Thought Language and Communication Index (TLC).

**Results:**

The clinical profiles of the PFTBI group met symptom/course criteria for: schizophrenia (n = 6), schizoaffective (n = 2), schizophreniform (n = 1), and paranoid psychosis (n = 1). PFTBI patients had a significantly reduced PANSS negative score relative to schizophrenia patients. No other significant differences between PFTBI and schizophrenia clinical profiles were found.

**Discussion:**

The clinical profile of PFTBI appears to be comparable to schizophrenia/ schizoaffective disorder except with respect to negative psychotic symptoms. Reduced negative symptoms in PFTBI have previously been reported in a small number of case studies, and thus warrant further investigation as a diagnostic distinction in this patient group.

